# Medicare immunosuppressant coverage and access to kidney transplantation: a retrospective national cohort study

**DOI:** 10.1186/1472-6963-12-254

**Published:** 2012-08-16

**Authors:** Vanessa Grubbs, Laura C Plantinga, Eric Vittinghoff, Ann M O’Hare, R Adams Dudley

**Affiliations:** 1 Division of Nephrology, University of California San Francisco, San Francisco General Hospital, San Francisco, CA, USA; 2 Department of Epidemiology, Emory University Rollins School of Public Health, Atlanta, GA, USA; 3 Department of Epidemiology & Biostatistics, University of California San Francisco, San Francisco, CA, USA; 4 University of Washington, Nephrology and Veterans Administration Puget Sound Healthcare System, Seattle, WA, USA; 5 Pulmonary and Philip R. Lee Institute for Health Policy Studies, University of California San Francisco, San Francisco, CA, USA

## Abstract

**Background:**

In December 2000, Medicare eliminated time limitations in immunosuppressant coverage after kidney transplant for beneficiaries age ≥65 and those who were disabled. This change did not apply to younger non-disabled beneficiaries who qualified for Medicare only because of their end-stage renal disease (ESRD). We sought to examine access to waitlisting for kidney transplantation in a cohort spanning this policy change.

**Methods:**

This was a retrospective cohort analysis of 241,150 Medicare beneficiaries in the United States Renal Data System who initiated chronic dialysis between 1/1/96 and 11/30/03. We fit interrupted time series Cox proportional hazard models to compare access to kidney transplant waitlist within 12 months of initiating chronic dialysis by age/disability status, accounting for secular trends.

**Results:**

Beneficiaries age <65 who were not disabled were less likely to be waitlisted after the policy change (hazard ratio (HR) for the later vs. earlier period, 0.93, p = 0.002), after adjusting for sociodemographic factors, co-morbid conditions, income, and ESRD network. There was no evidence of secular trend in this group (HR per year, 1.00, p = 0.989). Likelihood of being waitlisted among those age ≥65 or disabled increased steadily throughout the study period (HR per year, 1.04, p < 0.001), but was not clearly affected by the policy change (HR for the immediate effect of policy change, 0.93, p = 0.135).

**Conclusions:**

The most recent extension in Medicare immunosuppressant coverage appears to have had little impact on the already increasing access to waitlisting among ≥65/ disabled beneficiaries eligible for the benefit but may have decreased access for younger, non-disabled beneficiaries who were not. The potential ramifications of policies on candidacy appeal for access to kidney transplantation should be considered.

## Background

Because demand for donor kidneys exceeds supply several-fold [1], transplant centers are forced to make difficult decisions about who should receive transplantable organs. Central to the long-standing debate on how to allocate this scarce resource is the concept of equity---equal utilization of resources for equal need [2]---as women, the poor, and racial/ethnic minorities have historically been less likely to receive transplants than men, the wealthy, and non-Hispanic whites [3].

Health inequities may be influenced by policy [4]. When the Medicare end-stage renal disease (ESRD) program was established in 1973 rates of allograft survival were quite low. Therefore, immunosuppressant coverage created by the Omnibus Reconciliation Act of 1986 was limited to 1 year and incrementally extended to 3 years by mid-1995. In an effort to alleviate concerns of senior citizens being unable to afford their medications, the Beneficiary Improvement and Protection Act (BIPA) was passed in December 2000 to further extend coverage to lifetime—but only for Medicare beneficiaries whose eligibility was based on age or having a disability other than end-stage renal disease (ESRD) [5].

Several studies have shown that transplant recipients are at increased risk of medication noncompliance and subsequent graft loss if they are not able to afford their medications [6-8] and that incremental extensions in immunosuppressant coverage have been shown to have a positive effect on graft survival. Woodward and colleagues showed that, while graft survival at 3 years post-transplant was significantly lower for low- vs. high-income kidney transplant recipients when coverage was provided for only 1 year, there were no differences in 3-year graft survival by income when coverage was extended to 3 years [9]. They found similar improvements in graft survival among low-income recipients eligible for lifetime coverage [10].

Given the association between graft survival and immunosuppressant coverage, it is possible that Medicare immunosuppressant policy may also impact “upstream” care processes such as listing for transplant. ESRD patients without sufficient insurance coverage or financial resources may be viewed as less ideal transplant candidates and, therefore, may be less likely to be waitlisted. Though older patients with ESRD and those with extra-renal co-morbidities have historically been considered less ideal candidates than younger patients and those with fewer co-morbidities because of perceived surgical risk and decreased transplant and patient survival [11-19], the extension of lifetime immunosuppressant coverage to this group by BIPA may have increased their candidacy appeal. Therefore, we hypothesized that expansion of immunosuppressant coverage would differentially impact access to waitlisting for kidney transplantation depending on eligibility for it: with access increasing among older/disabled beneficiaries who would always have insurance coverage for immunosuppressants, but decreasing among younger/non-disabled beneficiaries who might not be able to afford their medications when Medicare eligibility ended at three years post transplant and, therefore, be at risk of rejecting a scarce resource. We sought to test this hypothesis in a population of Medicare beneficiaries who initiated dialysis within the years spanning the policy change.

## Methods

### Study sample

Using standard analysis files obtained with permission from the United States Renal Data System (USRDS), a national ESRD registry, we assembled a cohort of Medicare beneficiaries between the ages of 21 and 75 years who initiated chronic dialysis between 1/1/96 and 11/30/03. Individuals under 21 years were excluded from the analysis because children are subject to a different selection algorithm for kidney transplant [20] and those under 21 may have insurance coverage through their parents. Individuals over 75 years were excluded because this group receives <1% of all kidney transplants [13,21]. Beneficiaries who were waitlisted prior to initiating dialysis were excluded because preemptive waitlisting requires resources and early nephrology referral—factors that vary by insurance status, income, and race [22,23]---and our goal was to assess the policy’s effect among uniformly resourced cohorts.

We limited our study to patients with Medicare as their sole insurer using the *payer* variable at 90 days post-dialysis initiation in the USRDS Core Payer History file, which is compiled from a monthly record of payment source for ESRD service for each patient. Only those with Medicare as primary payer were included. Those with private insurance or dual coverage (Medicare secondary payer, group health organization, or other) were excluded because private forms of health insurance have variable drug coverage and Medicaid immunosuppressant coverage varies across states. Patients were followed starting at 90 days after initiation of dialysis because patients under age 65 whose only entitlement to Medicare is ESRD must wait 3 months on dialysis before becoming eligible for Medicare. The cohort thus did not include beneficiaries who died or received a kidney transplant within the first 90 days after onset of ESRD.

### Data source

The USRDS collects, analyzes, and distributes information on all treated ESRD patients in the United States. Data from the Patients file, the Medical Evidence file and Transplant and Payer History files were merged to create the final dataset. USRDS data also included participant ZIP code of residence, which we used to assign patients’ median ZIP code-level income according to the 2000 U.S. Population Census.

### Primary predictors

Our primary predictors were age/disability status and date of dialysis initiation. Disability was defined by employment status at time of dialysis initiation. Age and disability status were used to create a dichotomous age/disability status variable defined as age ≥65 years or disabled *or* age <65 years and non-disabled. Those who met Medicare eligibility for dialysis and were ≥65 years or disabled were eligible for the lifetime immunosuppressant benefit after 1/1/01.

### Outcome variables

The primary outcome was access to kidney transplantation, defined as time from day 91 after initiating chronic dialysis (initial Medicare eligibility for dialysis) to placement on the kidney transplant waitlist with censoring at death or at 12 months after initial Medicare eligibility for dialysis. Our 12-month time period is a reasonable time period for patients to become accustomed to dialysis and undergo kidney transplant evaluation. The time period is consistent with Healthy People 2020 objectives for chronic kidney disease and is slightly longer than that agreed upon by consensus in a study by Ayanian *et al.*[24].

### Covariates

Covariates included gender, race/ethnicity, income, primary cause of ESRD, co-morbid conditions at onset of ESRD, and ESRD network. We defined race/ethnicity as a categorical variable (non-Hispanic white (reference), non-Hispanic Black, Hispanic, or Other). We defined median ZIP code-level income in quartiles for the entire sample. Primary causes of ESRD as a 6-category indicator variable included diabetes (reference), hypertension, glomerular disease, cystic renal disease, other/unknown diagnosis. We adjusted our analyses using binary indicators (no (reference)/yes) for the following co-morbid conditions at the start of dialysis: congestive heart failure, ischemic heart disease, cerebrovascular accident, peripheral vascular disease, chronic obstructive pulmonary disease, tobacco abuse, drug dependence, and ability to ambulate. HIV without or with AIDS was defined as a three-level variable (no HIV/AIDS (reference)). We included a categorical variable for all but one of the 18 ESRD networks to account for geographic variability in access to renal transplant [25].

### Statistical analyses

We fit interrupted time series (ITS) Cox proportional hazard models to examine differential effects of the new coverage policy on access to kidney transplant by age/disability status, after adjusting for potential confounders. ITS is an established methodology that allows for different secular trends before and after the introduction of the new policy. This model accounts for the possibility that the intervention changes the trend, in which case simple pre-post comparisons can be misleading [26-29]. For each age/disability group, the full ITS model allowed for an abrupt change in the waitlisting rate at the beginning of 2001, as well as for different secular trends before and after the introduction of the new policy. If no statistically significant trends were found, we then simplified the model by including only the potential confounders. We assumed patients who initiated dialysis in 2000 and completed one year of follow-up in 2001 were affected by the new policy after 1/1/01; this was implemented using time-dependent covariates. Results of this model are summarized by plotting the estimated probability of being waitlisted within 12 months by group and date of dialysis initiation, accounting for censoring and holding all covariates constant at their sample means; these calculations combined information from the baseline survival function and adjusted relative hazard estimates. All analyses were implemented in Stata Version 12.0 (Stata Corp., College Station, TX).

## Results

**Figure 1:**
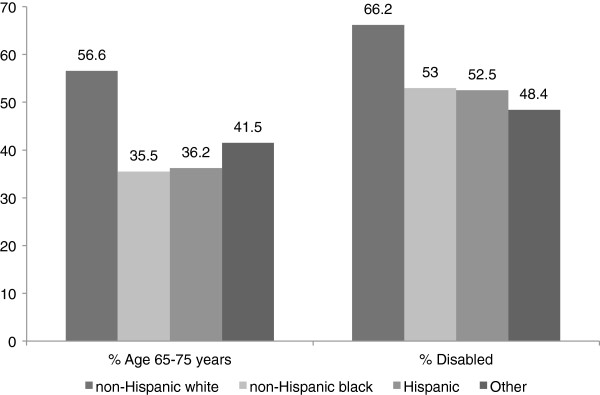
Age and disability status by race/ethnicity.

There were 241,150 beneficiaries who met our study inclusion criteria and were included in the analysis. Of these, 76,228 (31.6%) were age <65/non-disabled and 164,922 (68.4%) were age ≥65/disabled. Patient characteristics by age/disability status are shown in Table 1. Those age ≥65/disabled were more likely to be non-Hispanic white (Figure 1), live in a high-income ZIP code, and have higher prevalent co-morbidities than their age <65/non-disabled counterparts. The absolute percentage of those waitlisted was higher among age <65/non-disabled patients than among the age ≥65/disabled patients.

**Table 1:** **Patient characteristics by age/disability status**^**a,c**^

	**< 65/Non-disabled N = 76,228 (31.6%)**	**≥65/Disabled N = 164,922 (68.4%)**
Waitlisted (n,%)	7,199 (9.4)	6,062 (3.7)
Mean age, years (SD)	50.5 (11.1)	65.0 (9.6)
Male (n,%)	39,808 (52.2)	87,910 (53.3))
Race/Ethnicity (n,%)		
Non-Hispanic white	29,042 (38.1)	94,249 (57.1)
Non-Hispanic black	31,191 (40.9)	46,000 (27.9)
Hispanic	12,140 (15.9)	18,974 (11.5)
Other	3,855 (5.1)	5,699 (3.5)
Income quartile		
>75^th^	18,451 (24.2)	47,156 (28.6)
50-75^th^	17,279 (22.7)	40,734 (24.7)
25-50^th^	18,383 (24.1)	40,029 (24.3)
<25th	22,115 (29.0)	37,003 (22.4)
Primary Cause ESRD^b^ (n,%)		
Diabetes	37,345 (49.0)	89,971 (54.6)
Hypertension	17,505 (23.0)	39,780 (24.1)
Glomerular	7,724 (10.1)	11,118 (6.7)
Cystic	1,569 (2.1)	2,448 (1.5)
Other/Unknown	12,085 (15.9)	21,605 (13.1)
Co-morbid Conditions (n,% with condition)		
Congestive heart failure	18,501 (24.5)	61,259 (37.3)
Ischemic heart disease	10,961 (14.5)	48,848 (29.8)
Cerebrovascular accident	4,673 (6.2)	19,178 (11.7)
Peripheral vascular disease	7,634 (10.1)	30,571 (18.6)
Chronic obstructive pulmonary disease	3,346 (4.4)	15,508 (9.4)
Tobacco abuse	6,088 (8.1)	9,693 (5.9)
Drug dependence	2,014 (2.7)	842 (0.5)
Cancer	1,994 (2.6)	9,880 (6.0)
HIV no AIDS	822 (1.7)	481 (0.4)
HIV with AIDS	1,383 (2.8)	2,408 (2.2)
Non-ambulatory	1,964 (2.6)	7,504 (4.6)

^a^p < 0.001 for all characteristics.

^b^ESRD = end-stage renal disease.

^c^ESRD networks not shown.

**Figure 2:**
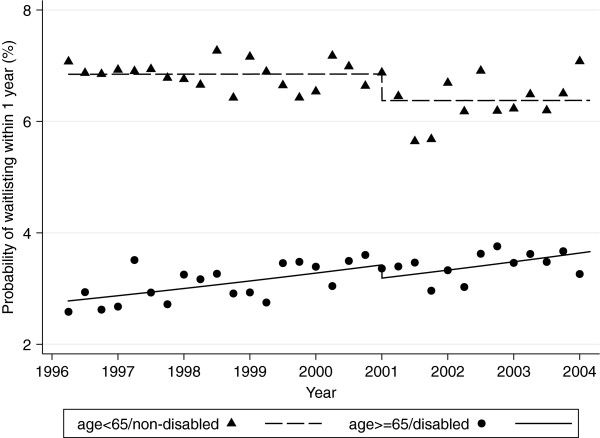
** Probability of waitlisting before and after policy change (in 2001) by age/disability status and quarter, adjusted for covariate means.** Lines represent estimated trends from final model of interrupted time series analysis.

ITS analysis revealed that changes in the likelihood of waitlisting over the study period differed significantly between the age <65/non-disabled and age ≥65/disabled groups (p < 0.001). However, as shown in Table 2, there was no evidence for changes in the slope for likelihood of waitlisting at the time of policy change (1/1/01) *within* either group in the full ITS model (both p > 0.350). In an intermediate model, which included group-specific changes in likelihood of waitlisting at policy change and overall secular time trends across the entire study period from January1998 through November 2003, there was no evident trend in the <65/non-disabled group (HR per increase of 1 year, 1.00, 95% CI 0.98-1.02, p = 0.989). In the final model, which omitted this non-significant time trend, we found a substantial systematic decrease in likelihood of waitlisting at the beginning of 2001 in the <65/non-disabled group (HR for the immediate effect of policy change, 0.93, 95% CI 0.89-0.97, p = 0.002). In the ≥65/disabled group, we found strong evidence for an increasing overall trend throughout the study period (HR 1.04 per year, 1.02-1.07, p < 0.001). There was a slight downward, but non-statistically significant, change in the probability of waitlisting at the beginning of 2001 (HR for the immediate effect of policy change, 0.93, 0.85-1.02, p = 0.135), after accounting for the significant time trend in the ≥65/disabled group. The adjusted probability of waitlisting by age/disability status and quarter, as well as the time trends based on the final model, are shown in Figure 2.

**Table 2:** Hazard ratios (HR) of waitlisting before policy change, after policy change (in 2001), and for the pre-/post-difference in waitlisting, by age/disability status and year

		**Secular trend**					**Pre-post difference**		
**Model**	**Group**	**Period**	**HR**	**95% CI**	**P-value**	**P#**^**b**^	**HR**	**95% CI**	**P-value**
**Full ITS**^**a**^**Model**	<65/non-disabled	before policy change	1.00	0.97-1.02	0.681	0.364	0.91	0.83-1.00	0.063
		after policy change	1.02	0.98-1.06	0.418				
	≥65/disabled	before policy change	1.05	1.02-1.08	<0.001	0.515	0.94	0.85-1.04	0.220
		after policy change	1.03	0.99-1.08	0.123				
**Intermediate Model**	<65/non-disabled	overall	1.00	0.98-1.02	0.989	-	0.93	0.85-1.01	0.099
	≥65/disabled	overall	1.04	1.02-1.07	<0.001	-	0.93	0.84-1.02	0.135
**Final Model**	<65/non-disabled	-	-	-	-	-	0.93	0.89-0.97	0.002
	≥65/disabled	overall	1.04	1.02-1.07	<0.001	-	0.93	0.84-1.02	0.135

^a^ITS = interrupted time series.

^b^P# = P-value for the equality of the within-group trends before and after policy change.

Those who were non-Hispanic black (vs. non-Hispanic white), were female, or lived in a ZIP code-defined area with lower income were less likely to be waitlisted (Table 3). Additionally, those with comorbid illnesses or disability were less likely to be waitlisted than their counterparts without these conditions. Waitlisting was more likely (up to 3-fold) among those with a primary cause of ESRD other than diabetes.

**Table 3:** **Hazard ratios (HR) of covariates, final model**^**a**^

	**HR**	**95% CI**	**P-value**
Gender			
Male	1.0		
Female	0.75	0.72-0.78	<0.001
Race/ethnicity			
Non-Hispanic white	1.0		
Non-Hispanic black	0.82	0.78-0.86	<0.001
Hispanic	1.16	1.09-1.22	<0.001
Other	1.25	1.16-1.35	<0.001
ZIP code-level income quartile			
>75^th^ percentile	1.0		
50-75^th^ percentile	0.90	0.86-0.95	<0.001
25-50^th^ percentile	0.85	0.81-0.89	<0.001
<25^th^ percentile	0.72	0.68-0.76	<0.001
Primary cause of ESRD			
Diabetes	1.0		
Hypertension	1.28	1.22-1.34	<0.001
Glomerular	2.50	2.38-2.62	<0.001
Cystic	2.99	2.75-3.24	<0.001
Other	1.37	1.29-1.45	<0.001
Unknown	1.46	1.33-1.61	<0.001
Co-morbid conditions^b^			
Congestive heart failure	0.59	0.56-0.62	<0.001
Ischemic heart disease	0.68	0.64-0.72	<0.001
Cerebrovascular accident	0.60	0.54-0.65	<0.001
Peripheral vascular disease	0.68	0.63-0.73	<0.001
Chronic obstructive pulmonary disease	0.47	0.42-0.53	<0.001
Tobacco abuse	0.84	0.78-0.91	<0.001
Drug dependence	0.35	0.29-0.39	<0.001
Cancer	0.33	0.29-0.39	<0.001
HIV no AIDS	0.09	0.04-0.18	<0.001
HIV with AIDS	0.65	0.54-0.78	<0.001
Non-ambulatory	0.32	0.26-0.39	<0.001

^a^ESRD networks not shown.

^b^Reference = no condition.

## Discussion

We expected that the most recent extension of Medicare immunosuppressant coverage would increase waitlisting among older/disabled beneficiaries who would always have insurance coverage for immunosuppressants, but would decrease waitlisting among younger/non-disabled beneficiaries who might not be able to afford their medications when Medicare eligibility ended at 3 years post transplant. After accounting for existing secular trends in waitlisting in a large national database, we found that the policy change did not appear to affect the already increasing access to the kidney transplant waitlist for the older/disabled beneficiaries, but did appear to decrease access for the younger/non-disabled beneficiaries.

While there is no absolute limit to the number of candidates who can be placed on the kidney transplant waitlist, the waitlist is restricted to those candidates transplant centers deem appropriate to receive the limited supply of transplantable organs. Historically, older kidney transplant candidates and those with extra-renal co-morbidities have been considered less ideal transplantation candidates, due to presumed reduced post-transplant functioning and survival [11-18]. Because transplant recipients who are unable to afford their medications are at increased risk of medication noncompliance and subsequent graft loss [6-8], it is conceivable that the extension of lifetime immunosuppressant coverage to ≥65/disabled Medicare beneficiaries would improve this group’s transplant candidacy appeal but would lessen that of younger/non-disabled patients with limited drug coverage, despite generally better overall health status. In other words, in anticipation of which patients will best care for the limited pool of deceased donor kidneys, transplant programs may be more inclined to waitlist older/disabled Medicare beneficiaries since the implementation of lifetime immunosuppressant coverage, but less inclined to waitlist the younger/non-disabled candidates who would lose Medicare coverage 3 years after transplant. Our findings of decreased waitlisting among the younger/non-disabled group after the policy change and increasingly lower likelihood of waitlisting with lower area income category support this assertion. This assertion is further supported by a recent study finding that most (67.3%) U.S. transplant programs report that they frequently or occasionally do not waitlist patients who are perceived to be unable to afford their immunosuppressant medications [30].

Historically, the explicit rationale for differentiating between those with and without existing Medicare eligibility was that those under age 65 and disabled only because of ESRD were expected to return to work because successful transplant was considered ‘rehabilitation’ [31]. However, return to work is uncommon post-transplant, especially among recipients not working prior to transplant, due to functional limitations and poor health status [32,33]; and finding work that provides private insurance is even less common [34].

Further, legislation for immunosuppressant coverage was established when post-transplant drugs were considered by some to be experimental, and 1-year graft survival was only 40% [35]. Now that renal transplant 5-year survival is upward of 80% and is associated with better quality of life than dialysis, transplantation has become the preferred treatment for ESRD [36]. Several studies have shown that, despite the significant cost of transplantation itself, post-transplant care and immunosuppressants, transplantation is more cost-effective than dialysis [5,35,36]. While Medicare spends a total of $73,008 and $53,446 per patient year of hemodialysis and peritoneal dialysis, respectively, only $24,572 per patient year is spent on kidney transplant [37]. Because Medicare resumes dialysis payments after transplant graft failure---which may be precipitated by limited coverage for immunosuppressant medications---current policy may be creating greater expense for the Medicare program through excess return to dialysis among <65/non-disabled patients. Our findings should be considered within the context of cost-effectiveness for the Medicare program, the primary payer for dialysis and transplantation in this country, and as part of the ongoing health care reform debate.

Because racial/ethnic minority ESRD patients are disproportionately younger and non-disabled than non-Hispanic whites, minorities may more often suffer decreased access under current policy. We found that non-Hispanic blacks in particular (but not other minorities) were less likely to be waitlisted than non-Hispanic whites. As evidenced in our study, racial/ethnic minorities are more likely to develop ESRD at a younger age than their non-Hispanic white counterparts [38-40] and are therefore further from aging into Medicare eligibility.

### Limitations

Our study is not without limitations. Disability status was determined from the employment status variable. Information regarding underlying cause of disability was not available. Therefore, we were not able to differentiate between disability status due to ESRD and disability for another reason, which may have significantly inflated proportions truly eligible for unlimited immunosuppressant coverage fulfilled by the criterion disability not due to ESRD. This misclassification may have been most pronounced among minority patients given their younger age at ESRD onset, increasing the likelihood that their disability was ESRD-related. However, the likely effect of this misclassification would be to attenuate the observed relationship between policy change and the likelihood of waitlisting among ≥65/disabled versus <65/non-disabled patients. Thus, the true impact of the policy on access to transplantation may be underestimated. Additionally, some patients may have obtained private insurance after 90 days of initiating dialysis (when we defined insurance status); however, we expect this is very low in our 12-month observation period given the “pre-existing condition” of end-stage renal disease, which likely excluded many patients at that time from affordable private insurance plans.

It is also possible that factors other than the policy change are also contributing to the observed trends in waitlisting of the ≥65/disabled population compared to the <65/non-disabled population. For example, provider attitudes regarding an upper age limit for transplantation could have changed over time in favor of older applicants, independent of the availability of lifetime immunosuppressant coverage. Also, the implementation of policy for acceptance of expanded criteria donor (ECD) kidneys in late 2002 may have also played a partial role in observed rates [41]; however, there was only a 15% increase in the number of ECD transplants [42] during this study period, and not all ECD kidneys go to the ≥65/disabled population [43]. Anticipation of the policy change could also be contributing to the steady increase over time in likelihood of waitlisting for the ≥65/disabled group---a finding that may reflect increasing patient and provider awareness of the policy and its ramifications. Such possibilities could not be examined here, given the limitations of the data, but future studies examining more recent trends in waitlisting, as well as reasons for waitlisting or not waitlisting potential transplant candidates, may help elucidate underlying causes.

Lastly, we recognize that waitlisting is but one step in access to kidney transplantation. Many factors, such as blood type and pre-formed antibodies, are important determinants of receiving a kidney transplant. Because these factors are only routinely measured in waitlisted candidates, they were largely missing in our dataset, rendering us unable to test our hypothesis in the entire ESRD population.

## Conclusions

In summary, our study suggests that the most recent Medicare coverage extension did not appear to change the already increasing access to the kidney transplant waitlist for the ≥65/ disabled beneficiaries eligible for the benefit but may have decreased access for the <65/non-disabled beneficiaries who were not. Therefore, the potential ramifications of policies on candidacy appeal for access to kidney transplantation should be considered.

## Competing interests

The authors declare they have no competing interests.

## Authors’ contributions

VG acquired the data, participated in study design and statistical analysis and interpretation of the data, and drafted the manuscript. LCP and EV participated in statistical analysis and interpretation of the data and critical revision of the manuscript. AMO and RAD participated in study design and critical revision of the manuscript. All authors have read and approved the final manuscript.

## Disclaimer

The data reported here have been supplied by the United States Renal Data System (USRDS). The interpretation and reporting of these data are the responsibility of the author(s) and in no way should be seen as an official policy or interpretation of the U.S. government.

## Funding/ Support

VG was supported by National Institutes of Health/ National Institute of Diabetes and Digestive and Renal Diseases Diversity Supplement to R01 DK70939 and by the Harold Amos Medical Faculty Development Program of the Robert Wood Johnson Foundation. RAD was supported by an Investigator Award in Health Policy from the Robert Wood Johnson Foundation.

## Pre-publication history

The pre-publication history for this paper can be accessed here:

http://www.biomedcentral.com/1472-6963/12/254/prepub
